# Detection of Nitrated, Oxygenated and Hydrogenated Polycyclic Aromatic Compounds in Smoked Fish and Meat Products

**DOI:** 10.3390/foods11162446

**Published:** 2022-08-13

**Authors:** Elisa Sonego, Bina Bhattarai, Lene Duedahl-Olesen

**Affiliations:** 1 Department of Chemistry, University of Rome “La Sapienza”, Piazzale Aldo Moro, 5, 00185 Rome, Italy; 2 National Food Institute, Technical University of Denmark, Kemitorvet, DK-2800 Lyngby, Denmark

**Keywords:** GC-QTOFMS, simultaneous detection (OPAH, NPAH, PAH 4), method validation, identification

## Abstract

Polycyclic aromatic hydrocarbons (PAHs) are present in smoked food products. More toxic nitrated (NPAH) and oxygenated (OPAH) PAHs derivatives are found concomitantly to PAHs and are therefore believed to be found in smoked food products. However, only a few PAH analyses on food include these derivatives. We adjusted and successfully validated a GC-QTOFMS method including 13 NPAHs and 2 OPAHs as well as the 4 regulated PAHs for analysis of 14 smoked (13 fish and one bacon) and one pan fried fish samples.OPAHs were detected in the highest concentrations in 13 of 15 samples. Non-target screening revealed the presence of an additional four OPAHs and two methylated PAHs. Future food analysis should, based on these results, focus on PAH and oxygenated derivatives.

## 1. Introduction

**Figure 1:**
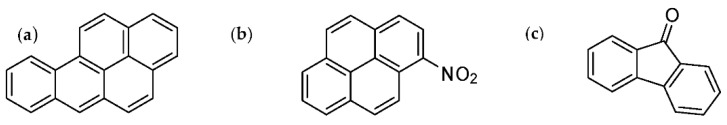
Examples of chemical structures of polycyclic aromatic hydrocarbons (PAHs) (**a**) benzo[*a*]pyrene, PAH, (**b**) 1-nitropyrene, nitrated derivative (**c**) 9-fluorenone, oxygenated derivative.

Polycyclic aromatic hydrocarbons (PAHs), aromatic compounds composed of carbon and hydrogen atoms in two or more fused benzene rings, are ubiquitous in the environment. More than hundred PAHs and their derivatives, e.g., methylated, nitrated and oxygenated, have been found. PAHs are formed mainly by human activity and can be found in food, e.g., food exposed to incomplete combustion of organic matter [1]. Incomplete combustion of, e.g., wood for the smoking of food, has also been found to result in the formation of PAHs derivatives [2,3]. PAHs derivatives such as nitrated and oxygenated polycyclic aromatic hydrocarbons have at least one hydrogen atom substituted by a nitro-group (NPAHs) or an oxygen atom (OPAHs) (examples in Figure 1). Formations of NPAHs have been reported due to the chemical reactions of PAHs in the atmosphere with dinitrogen pentoxide (N_2_O_5_), nitrogen dioxide (NO_2_) and nitrogen trioxide (NO_3_) radicals [4], whereas photochemical oxidation of PAHs or biological transformation has been shown to lead to the formation of OPAHs [5,6].

The main exposure to PAHs for non-smokers and/or non-occupationally exposed people is through the diet [7]. The contamination of PAHs in food occurs both via exposure to a polluted environment and during food processing such as smoking, drying or barbecuing [1,8,9]. It can be assumed that every food that contains PAHs also contains their derivatives [10,11,12].

Exposure to PAHs and their derivatives have deleterious effects. Several PAHs and their derivatives show carcinogenic activity and have been classified by the International Agency for Research on Cancer (IARC) (Table 1). The most toxic PAH compound studied is benzo[*a*]pyrene (BaP) that has been classified as being carcinogenic to humans (group 1), whereas a couple of PAHs and NPAHs have been classified as probable cancer-causing agents (group 2A) and several others have been classified as possible carcinogenic agents (group 2B). Additionally, OPAHs such as 9,10-anthraquinone (ATQ) have been classified as possible carcinogens (group 2B) [13,14], whereas the parent PAH anthracene belongs to IARC classification group 3 together with several other PAHs, indicating agents not classifiable as to its carcinogenicity to humans. Additionally, NPAHs have a higher risk of causing cancer illustrated by the IARC classification for, e.g., pyrene classified as group 3, whereas nitro- and dinitro-pyrenes are classified as group 2A and 2B compounds, respectively (Table 1). Since such PAHs derivatives, as already mentioned, are believed to be present in combination with the PAHs, the derivatives are assumed to pose a significant risk to human health and knowledge on their occurrence and concentrations in food is relevant.

Studies on PAH derivatives are mostly limited to atmospheric particulate matter and environmental samples [5,18,19,20,21]. However, the PAHs derivatives can, similar to PAHs, contaminate smoked products and contamination is believed to be dependent on applied smoking technology, type of wood, smoke generator and combustion temperature [3,22].

The current knowledge of the presence of NPAHs in food is limited to a few studies. The presence of 1-nitropyrene in smoked tea and 9-nitroanthracene in peated malt reported in 1984 was one of the first reports on NPAHs in food [2]. Later, 1-nitropyren was detected in grilled chicken due to incomplete combustion of fat into pyrene, in the presence of nitrogen dioxide during cooking [23] and several nitrated or nitrogenated PAHs as well as oxygenated PAHs were tentatively identified in barbecued sausages [24]. NPAHs have thereafter been analysed in grilled and smoked meat and fish [3,10,11,12,25,26], coffee [25,26], tea [27], beer [28], cereals and vegetables [29], as well as oil, spices and cheese [2,30,31]. As regards to OPAHs, they were similarly found in beverages such as:, coffee [25], tea [27], beer [28], milk and milk powders [32]. The OPAHs were also detected in oils [33,34,35,36,37], fried products such as peanuts [38], bread [39,40] and barbecued or smoked foods [3,22,24,41]. 

Methods for the analysis of PAHs have often been extended for the analysis of OPAHs after minor modifications [3,22,35]. On the other hand, for the NPAHs determination, single specific analytical methods has previously been developed [10,11,12,31,42,43,44,45,46]. Only few studies included simultaneous sample results on PAHs, NPAHs and OPAHs [3,24,28].

To ensure consumer protection a maximum limit of PAHs, namely for benzo[*a*]pyrene and the sum of benzo[*a*]pyrene, benz[*a*]anthracene, chrysene and benzo[*b*]fluoranthene (PAH4) has been set in certain foods in Europe in Commission Regulation No. 1881/2006 with amendments [47]. Despite the evidence about the PAHs derivatives adverse effect for human health, ATQ is the only PAH derivative regulated in European Union with maximum levels in meat, milk, wheat and tea in Commision Regulation No 1146/2014 [48].

We present results from simultaneous analysis of real-life samples for PAHs, NPAHs and OPAHs together with method validation. The optimized method allows quantitative analysis of PAH4, NPAHs and OPAHs simultaneously by the use of a unique sample treatment protocol and instrumental injection in order to simplify the screening of these harmful derivatives and collect more data for future risk assessment. In addition, we include non-targeted screening for PAHs and their derivatives for which reference substances were not available.

## 2. Materials and Methods

### 2.1. Chemicals and Materials

Native and deuterated PAHs standards were purchased as a separated mixture of 16 PAHs and 16 deuterated PAHs at 10 ng/µL in cyclohexane from Dr. Ehrenstorfer (LGC Standards, Wesel, Germany). 9FLO and ATQ (full forms are presented in Table 1) were obtained as powder by Sigma-Aldrich (Darmstadt, Germany), 4NPyr powder was purchased from LCG Standards (Wesel, Germany). The NPAHs (1NNap, 2NNap, 5NAce, 2Nflu, 9NAnt, 9NPhe, 3NPhe, 3NFla, 1Npyr, 7NBaP, 6NChry, 1,3DNPyr, 1,6DNPyr, 1,8DNPyr, 6NaP) (full forms are presented in Table 1) were purchased from Accustandard (New Haven, CT, USA) as stock solutions at a concentration of 100 ng/mL in toluene. Deuterated NPAHs standards in toluene, 1-nitronaphthalene-d_7_ at 1000 µg/mL, 2-Nitrofluorene-d_9_, 9-Nitroanthracene-d_9_, 1-Nitropyrene-d_9_, 3-Nitrofluoranthene-d_9_ and 6-Nitrochrysene-d_11_ all 100 µg/mL were purchased from Chiron (Dandenong, Australia). Stock solutions were prepared in toluene and stored in the dark at −20 °C.

HPLC grade acetone, ethyl acetate, n-pentane and cyclohexane were from Rathburn Chemicals Ltd. (Caberston Road, Walkerburn, Scotland), HPLC grade n-hexane and toluene as well as sodium sulfate (s) were from Merk (Darmstadt, Germany). Hydromatrix was from Agilent Technologies (Santa Clara, CA, US), Ottawa sand was purchased from Fisher Scientific (Kamstrupvej, Roskilde, Denmark), VWR paper filter 516-0314 were obtained from VWR (Radnor, PA, US). The gel permeation chromatography (GPC) was performed using a 500 × 40 mm packed with Bio Beads, S-X3, 200–400 mesh obtained from Bio-Rad Laboratories (Hercules, CA, USA). Minisart, non-sterile PVDF 450 mm with membrane 0.45 µm syringe filters from Sartorius (Göttingen, Germany) and SPE silica column (Isolute, 500 mg, 3 mL) were obtained from Biotage (Uppsala, Sweden).

### 2.2. Samples

Eleven smoked fish samples, two salmon, two herring, two mackerel and one halibut from fishmongers in Kongens Lyngby, Denmark and two salmon, one mackerel and one halibut purchased in different retail markets as shelf products were all purchased in the period July–September 2021. One traditional hot smoked Polish bacon sample was obtained from a charcuterie in Poland (Gdańsk). Raw hake and cod fillets from a retail market in Kongens Lyngby were used for method validation and smoking process studies, respectively. Prior to method validation, raw hake fillets were baked in the oven at 180 °C for 10 min to obtain similar muscle structure as for smoked samples. All the samples were stored at −20 °C.

Prior to homogenization of all frozen samples, all skin and bones were removed to minimize the fat loss of the contact surfaces. Frozen samples were cut into small pieces, homogenized using Waring commercial blender (Bie & Bernsten A.S, Denmark) and weighed for further processing as 5.0 g aliquots. A detailed description of the samples and their fat content is reported in the Supplementary Material (Table S1). Fat content was determined gravimetrically at 70 °C after accelerated solvent extraction (ASE) as described by Lund and coworkers [49].

### 2.3. The Smoking Experiments

Formation of PAH4, NPAHs and OPAHs under different processing conditions were tested with cod fillet which was pan fried (non-smoked reference) and smoked on a gas barbecue (Napoleon, Rouge) with smoke from hickory wood chips (weber) at 200 °C (experiment 1) and 360 °C (experiment 2). The wood used to smoke the samples was soaked in cold water for 1 h, according to the producer instructions before use.

### 2.4. Sample Treatment

An inhouse PAH method [50] was applied with modifications. In short, extraction with n-hexane: acetone (3:1 *v*/*v*) by pressurized liquid extraction was performed in 66 mL stainless steel vessels (cells, Dionex) containing two cellulose filters (Type D28) and 1 g of hydromatrix using a Dionex ASE 350 (Sunnyvale, CA, USA). Samples of 5 g were mixed in a mortar with 5 g of Ottawa sand (Fisher Scientific), previously dried at 400 °C for 3 h, and 10 g hydromatrix. Samples were fortified with internal standards and covered with Ottawa sand. The ASE extraction was performed with two cycles at 100 °C and 1500 psi with a static hold of 5 min and a flush volume of 75%. The extract of approximately 100 mL was filtered through a paper filter with 25 g of sodium sulphate.

Modified evaporation was carried out in a water bath at 35 °C with a gentle nitrogen flow (0.5 mL/min) until 1–2 mL. Filtrate (0.45 µm polytetrafluoroethylene, PTFE filter) was cleaned up to remove the fats by GPC (S-X3) and cyclohexane:ethyl acetate 1:1 (*v*/*v*) elution on an automated Gilson ASPEC XL system (Gilson, France), as described by Fromberg and co-workers [51].

To avoid fat overload on the GPC column, samples were diluted with cyclohexane:ethylacetate 1:1 *v*/*v*) to 5, 10 or 20 mL (fat content of <17.5%, 17.5–35% and >35%, respectively) and loaded with 5 mL injections onto the GPC column. The purified extract was concentrated to 1 mL and submitted to SPE (3 mL, 500 mg Si) purification with 3 mL cyclohexane conditioning, followed by sample loading and elution with 3 mL of cyclohexane. Cleaned samples were evaporated to 200 µL at 35 °C using a gentle nitrogen flow, transferred to a GC vial equipped with a pointed bottom with 100 µL of toluene as the solvent keeper. The sample was concentrated to 100 µL and then analysed by GC-QTOFMS.

### 2.5. GC-QTOFMS Analysis and Quantification

The GC-QTOFMS analysis was performed using an Agilent 7200 Accurate-Mass Quadrupole Time-of-Flight (QTOF) GC/MS system. The instrument was equipped with a programmed temperature vaporization (PTV) injector. Then, 4 μL was injected at 50 °C in solvent-vent mode; the temperature was held for 1 min before a temperature increase at a rate of 480 °C/min to 290 °C held for 2.2 min, followed by an increase to 330 °C (by a rate of 720 °C/min) kept for 10 min.

The chromatographic separation was performed by coupling J&W Select PAH capillary column (Agilent Technology Santa Clara California USA, 15 m × 150 μm × 0.1 μm) with a HP5MS ultra inert capillary columns (Agilent Technology, 15 m × 250 μm × 0.25 μm) with backflush between the columns and a Helium flow of 1.4 and 1.2 mL/min, respectively. The final program for the separation of NPAHs, OPAHs and PAH4 was a GC oven initially held at 70 °C for 3.3 min, gradually raised to 180 °C at 50 °C/min, further raised to 220 °C at 4 °C/min, followed by an increase to 280 °C at 10 °C/min, and held for 2 min and further increased to 300 °C at 20 °C/min, held at 6 min and finally held at 310 °C for 10 min after a heating rate of 14 °C/min. To prevent column contamination, a column backflush was performed after each run. The mass spectrometer operated in electron ionization mode with an electron energy of 70 eV and 230 °C and a mass range of *m*/*z* 50–500 and an acquisition range of 5 spectra/second.

**Figure 2:**
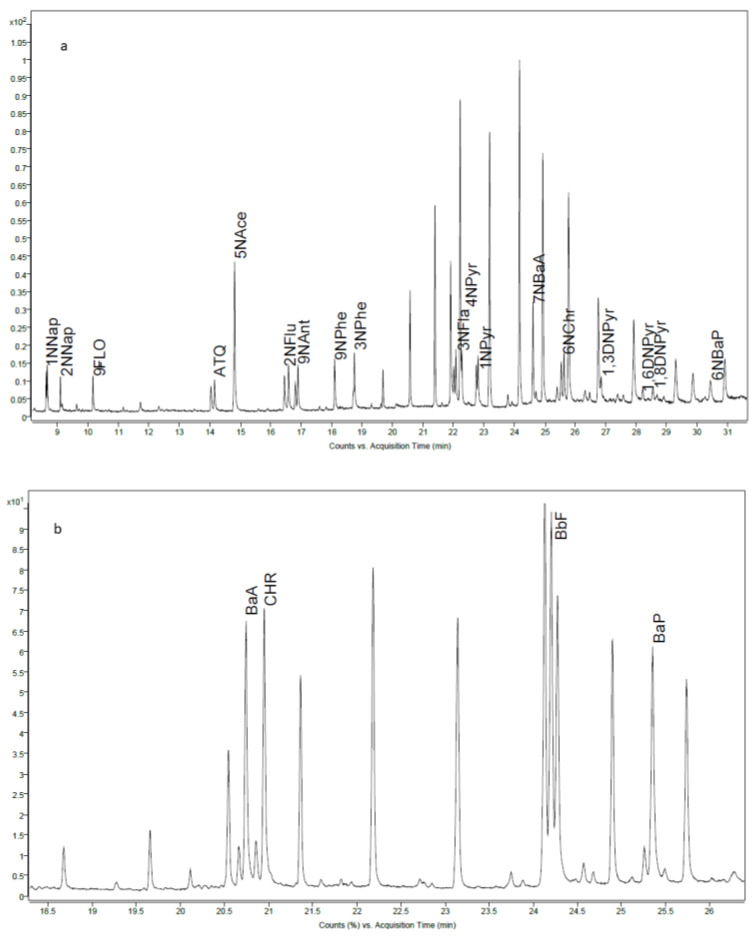
Chromatograms of standards (500 ng/mL) with quantified compounds labelled with corresponding compound name (**a**) NPAHs and OPAHs (**b**) PAH4.

Prior to analysis of NPAHs, OPAHs and PAH4, a single standard solution at a concentration of 500 ng/mL was injected to determine the retention time and to select the most abundant ions for each compound. Compound identification was performed based upon 3 digits accuracy *m*/*z* values and the retention time (Table 2). A chromatogram for a multi standard mix of NPAHs, OPAHs and PAH4 (500 ng/mL) can be found in Figure 2a,b.

Standard diluted solutions in the range 1–1000 ng/mL were injected to investigate the linearity range of compound responses and the instrumental sensibility for each PAHs derivative. The instrumental limit of quantification was evaluated by the signal to noise ratio of 6 ×S/N and found to be 10–20 ng/mL for all derivatives, except dintro-pyrenes (DNPyrs) with 100 ng/mL.

The internal standard method was, as mentioned, used for quantification. Since not all deuterated NPAHs and OPAHs were available, the closest in terms of retention time was used as the internal standard. Quantification was performed by the solvent-matched calibration curve in the concentration range 10–500 ng/mL and 5–500 ng/mL for PAH 4 and PAHs derivatives, respectively. A blank sample and a quality control (fortified at 5 µg/kg) were included in each analytical session and results outside the recovery range of 85–115% were corrected for the recovery. Quantification of the samples with signals out of the calibration range was performed after dilution.

### 2.6. Method Validation

A previously validated method for PAHs [50] was extended for this study to selected PAHs derivatives (Table 2) as well as PAH4. The optimized method was submitted to validation for the PAHs derivatives. The EC method performances criteria for PAH4 were followed as a guide for the PAHs derivatives. The validation was carried out at three different concentration levels with a requirement for at least six degrees of freedom for reproducibility according to ISO/IEC 17025:2017 accreditation.To evaluate the total method performances (PAHs method and modified method), evaporation temperature (35 °C or 40 °C) and evaporation method (rotor vaporisation or N_2_ flow) recovery experiments on spiked heated hake samples (5 µg/kg except for 10µg/kg for DNPyrs, 2NFlu and 5NAce) with triplicate analyses were performed. Internal standards were added post-extraction in all experiments.

The linearity of the optimized method was evaluated in terms of regression coefficient with a criterion of R^2^ value above 0.995. A matrix-matched calibration curve was obtained spiking five hake sample extracts with 10 µL of concentrated standard solutions. The matrix effect was evaluated by the ratio between the slope of the matrix-matched and the solvent-matched calibration curves multiplying by 100.

The oven heated hake was used for the method validation by spiking at three different concentration levels (2 µg/kg, 5 µg/kg and 10 µg/kg for 1NNap, 2NNap, 5NAce, 2Nflu, 9NAnt, 9NPhe, 3NPhe, 1Npyr, 7NBaP, 6NChry, 6NaP, 9FLO, ATQ and 4 µg/kg, 10 µg/kg and 20 µg/kg for 3NFla, 1,3DNPyr and 1,6DNPyr, 1,8DNPyr). The lower concentration level was chosen closer to the expected limit of quantification (LOQ) and the middle concentration level was also used as a quality control (QC) in each analytical session. For each spike level at least four replicates were made in two different analytical sessions at different days with two different operators (4 + 4).

The relative repeatability standard deviation (RSD_r_) and the relative intra-laboratory reproducibility standard deviation (RSD_R_) were calculated for each concentration level according to ISO 5725-2 [52]. The Horwitz Ratio (HorRat) was calculated for repeatability and intra-laboratory reproducibility (HorRat_r_ and HorRat_R_) using the modified Horwitz equation with the modified Horwitz RSD value of 22%. The total recoveries were calculated at each concentration level. The limit of detection (LOD) and LOQs were calculated according to ISO 5725-2 [52] using the standard deviation of 10 replicates spiked at the lowest level multiplied to three times and six times, respectively.

The official validation acceptance criteria for EU PAH4 regulation in foods [53] were used as a guideline for all PAHs derivatives validation, in particular a criterion of a recovery between 50 and 120% and HorRat_r_ and HorRat_R_ values < 2.

### 2.7. Non-Target Screening

The GC-QTOFMS data were analysed by the Agilent MassHunter Qualitative Analysis version B.07.00 (Agilent technologies Inc., Santa Clara, CA, USA). Deconvolution was performed using the Agile 2 algorithm with the following parameters: S/N: 20; Absolute height ≥ 100 counts; Absolute area ≥ 5000 counts; RT window size factor: 100; sharpness threshold 25%. The deconvoluted peaks were searched against W10N14 (Wiley 10 NIST 14 library) for identification with a match score of 80. The exact mass of the compounds were further confirmed using the MS Interpreter present in the NIST MS Search version 02.

## 3. Results and Discussion

### 3.1. Extraction Method and Instrumental Analysis Optimization

In this study, PAHs derivatives were included by optimizing the previous sample preparation method for the extraction of PAHs from fish [50] and the quantification of PAHs by GC-QTOFMS [54]. The original protocols differed from the procedure described here for the evaporation conditions and GC-QTOFMS parameters.

To assay the method performances for PAHs derivatives, the extraction yield (extraction recovery) of the original method was tested in triplicate and found to be 8–46% for a single PAH derivative and therefore not acceptable. The original method included a long evaporation step performed by rotatory evaporation at 40 °C to concentrate the ASE extract. Since losses of PAHs derivatives during evaporation were previously reported [31,55,56,57], evaporation recovery tests were performed in order to set better evaporation conditions.

Both rotatory vacuum evaporation and nitrcures (35 and 40 °C). The results obtained at the temperature of 40 °C resulted in the complete loss of DNPyrs and gave the worst results for all the analytes, with overall recovery for nitrogen flow at 48% and for rotatory vaporization at 63%. The evaporation obtained by nitrogen flow at 35 °C resulted in overall evaporation recoveries of 79% with a coefficient of variance (CV%) between 3 and 16% compared to rotatory evaporation recoveries of 64% and CV% between 3 and 30% (single results can be seen in Table S2). Irrespective of the sample preparation technique, 1,8 DNPyr had the highest CV%, resulting in low reproducibility. Nitrogen flow at 35 °C was chosen for all evaporation steps with the advantage to perform simultaneous evaporation of several samples compared to rotatory evaporation.

The optimized method had extraction recoveries with final triplicate acceptable extraction recoveries between 55 and 82% (except for 1,3 DNPyr (28%) and 1,8 DNPyr (20%)) and CV% well below 23%. In the literature, only a few methods are present for the simultaneous detection of PAHs, NPAHs and OPAHs, which mostly had a dedicated analytical method.

### 3.2. Validation Results

Solvent matched calibration curves showed linearity always associated with R^2^ > 0.995 and matrix-matched (prepared using a hake matrix) calibration curves provided R^2^ between 0.995 and 0.999. The matrix-matched calibration curves, resulted in recoveries of 85–115% (Table 2). Therefore, the effect of matrix was considered negligible, and the quantitative analysis was performed using the solvent-matched calibration curve.

The sample blank showed a slight contamination for 9FLO and ATQ; however, the signals were below LOQ and an order of magnitude lower compared to the positive samples, so the average value was subtracted to the sample results. The procedural blank also showed 9FLO and ATQ contamination, so for the recovery calculation it was taken into account.

The results of relative standard deviation for in-laboratory reproducibility and repeatability (mean, RSD and HorRat values) and the total recovery are reported for each concentration level (Table S3). Table 2 shows the mean values for RSD_R_ (intra-laboratory reproducibility), RSD_r_ (repeatability) and total recovery for three validated spike levels, followed by the calculated LOD and LOQ. For 1,3DNpyr and 1,6DNPyr, only values at concentration levels which passed the validation were included.

The repeatability (RSD_r_) were in the range 2–19% and HorRat_r_ < 2 were obtained, while the intra-laboratory reproducibility (RSD_R_) resulted in the range 3–18% and HorRat_R_ < 2, with the exception of DNPyrs. In particular, 1,3DNPyr, 1,6DNPyr and 1,8DNPyr did not give acceptable values for the medium and the higher spike level, and 1,8DNPyr neither for the lower.

The average total recoveries were acceptable for all PAHs derivatives within the range 88–107%, except for ATQ (64%) and DNPyrs (70%, 91%), the latter only passing overall acceptance criteria following EU regulation for PAH4 for 1,3DNPyr and 1,6DNPyr at the lowest validation level (EU regulation 333/2007 with amendments [53]). HorRat values above 2 for both medium and high validation levels for 1,3DNPyr and 1,6DNPyr resulted in only fulfilled validation criteria for one spike level.

The LOD and LOQ obtained for NPAHs and OPAHs were low and suitable for the food contamination analysis. LOD and LOQ ranged from 0.19 to 0.80 µg/kg and from 0.58 to 1.6 µg/Kg. Acceptance criteria in the European PAH4 regulation are 0.3 and 0.9 µg/kg, for LOD and LOQ, respectively.

### 3.3. Smoked Samples

The developed method was used to quantify 13 NPAHs, 2 OPAHs and 4 PAHs in 14 smoked and one pan fried samples. The presence of 1,8DNPyr was evaluated; however, it was not detected in any samples and therefore not included in Table 3.

The overall contamination contribution given by each group was calculated as a sum of NPAH, OPAH and PAH4. Not detected compounds (nd in Table 3) were included as zero and the values quantified between LOD and LOQ were used according to the EFSA left censored data management report [58], in order to provide the best use of the available data for future dietary exposure assessment. Only 5 NPAHs, namely 1NNap, 2NNap, 5NAce, 9NAnt and 4NPyr, were detected in real samples and only 1NNap and 9NAnt were above the LOQ, with a concentration of 5.6 µg/kg and 1.0 µg/kg in herring (HE1) and bacon (B1), respectively (Table 3). These results agreed with Deng et al. [44], who reported negligible contamination in smoked bacon for 1NNap, 2NFlu and 1NPyr (which were the NPAHs monitored). On the other hand, the OPAHs were detected in almost all samples, only one mackerel sample was free of both PAH and derivatives (M2). In general, our results showed higher concentrations of 9FLO compared to ATQ (overall mean values obtained were 20 µg/kg and 4.9 µg/kg, respectively). Smoked bacon (B1) and the cod smoked at high temperature (C4) had ATQ concentrations above the EU maximum limit of 10 µg/kg applicable in meat [48]. In addition, 9FLO concentrations were very high in the smoked bacon, the signal was out of the calibration range and the value reported must be considered indicative. The smoked bacon also had high concentrations of PAHs, with a BaP concentration of 5.3 µg/kg and the sum of PAH4 was 38 µg/kg, which exceeded the EU maximum limit for smoked fish and meat of 2 µg/kg and 12 µg/kg, respectively [48]. Conversely, between the fish samples, only five samples showed PAHs contamination with concentrations above the method LOD. Two mackerel samples (MA1 and MA2) had less than 0.7 µg/kg or no PAHs. Cold smoked Salmon (SA2 and SA3) had less than 3 µg/kg OPAHs, which is less than for hot smoked salmon (SA1 and SA4, 8.7 and 22 µg/kg, respectively). The results indicate processing differences since the results could not be explained by differences in fat content.

One of the frequently detected and reported NPAHs is 1NNap [30,42,44,45]. The concentration for 1NNap was reported from 2.0 to 162 µg/kg, where the highest concentration was reported for smoked samples [42,44]. These reported mean concentrations were similar to our result (Table 3). Conversely to us, the 1NPyr has also been commonly detected [10,11,23,42,45]. In comparison to our results, 2NFlu and 3NFla were not detected, for 2NFlu in twenty meat and fish samples [44] and for 3NFla in 11 barbecued samples [42] and smoked sausages [45]. Our method did not detect any di-nitro pyrenes (DNPyrs) in samples, which was conversely reported in eight of eleven barbecued foods in a concentration range of 3.2–13 µg/kg (1,8DNPyr, [42]).

Zastrow et al. studied the ATQ formation under different smoking conditions of sausages and reported slightly lower ATQ concentrations than ours in the range of 1.3–3.2 µg/kg [22]. Conversely to our outputs, Chen and co-workers reported that ATQ and benzanthrone was the dominant contaminant compared to 9FLO [3].

The samples obtained from the controlled smoking experiment are reported in the first three columns of Table 3. The pan-fried cod (C2) was not surprisingly free of PAH4 and NPAHs contamination. In spite of this, OPAHs were detected also in the pan-fried sample with a concentration of 4.9 and 2.9 µg/kg for 9FLO and ATQ, respectively. OPAHs are reported to be more bioaccumulative than their precursor and present also in raw fish [59]. Since the boiled cod (C1) also showed 9FLO and ATQ contamination at 3.4 and 2.3 µg/kg, it was assumed that the concentrations found were only due to an endogenous contamination of the fish meat itself. This also confirms that the boiling process does not result in the formation of PAHs or their derivatives. From this point of view, it could not be excluded that the fried cod contamination was also at least partially due to bioaccumulated 9FLO and ATQ. Regardless, the smoking experiments led to increased OPAHs and PAHs contamination (Table 3 C1 to C4). The cod smoked at 360 °C (experiment 2, sample C4) showed higher concentrations of OPAHs compared to smoking at 200 °C, both for 9FLO and ATQ, and the latter had a concentration above the regulated limit of 10 µg/kg. Conversely, the sum of PAH4 was comparable with 0.3 and 0.2 µg/kg for smoking experiment 1 (C3) and 2 (C4), respectively.

The contamination obtained from the smoking experiment 2 (C4) and of the smoked bacon (B1) confirmed that a higher temperature resulted in higher contamination of PAH derivates, especially in uncontrolled smoking processes.

In general, both our and the other few studies including PAHs and their derivatives showed that OPAHs concentrations were often higher than PAHs and NPAHs [3,22,41]. These results suggest broadening the investigation to more OPAHs derivatives, both because of the higher concentrations compared to PAHs and taking into account the increased unwanted effects of these compounds [60,61].

### 3.4. Non-Target Screening

In addition to the quantitative target analysis, a non-target approach (qualitative) was applied identifying the presence of additional seven PAHs, four OPAHs and two methylated PAHs. All the tentatively identified PAHs (for which no standards were available), were identified at a confidence level of 2a (according to Schymanski et al. [62]) for which a match with a library spectrum was present. Since NIST library contains mass spectrum with unit mass, a NIST MS interpreter was used to crosscheck the exact mass of the precursor ions and product ions. The MS fragments of the identified PAHs are presented in Table 4. Identification of structural PAH isomers, e.g., Xan and 2-OH-Flu, Phe and Ant, and Fla and Pyr, is challenging because they have same exact mass and similar MS fragmentation pattern. These groups of isomers were distinguished from each other based on the difference in the retention time and ion abundance of MS fragments.

Xan and 2-OH-Flu have the same exact mass and mass fragments, where the ion abundance of the fragment ions (*m*/*z* 76.031, 151.055, 152.063, 181.066, 184.088) were higher in Xan, which was in line with the data from NIST (MS interpreter). Phe and Ant were identified based on the difference in the retention time [63,64] and ion abundance. The ion abundance of the major ions *m*/*z* 176.062, 177.069, 152.062, 151.054 was higher in Phe, which was in line with the data from NIST (MS interpreter) and [65]. Similarly, Fla and Pyr were identified based on the difference in the retention time, as reported previously by [64]. The slight difference was observed in the ion abundance of some of the mass fragments, as observed previously by [66] and as in the data from NIST (MS interpreter). The *m*/*z* 100.032, 174.068, 201.071, 203.085, and 204.064 were higher in pyrene.

Between the four OPAHs (1-Ind-one, 4-Acy-ol, 2-OH-Flu, Xan), 1-Ind-one was detected in most of the (hot and cold) smoked samples, while the remaining three were detected in the hot smoked bacon, herring and mackerel sample.

Although no OPAHs were detected in Mackerel (MA1 and MA2 in Table 3) with the target analysis, the non-target approach revealed the presence of 1-Ind-one and Xan (Table 4).

According to Schlemitz and Pfannhauser [10,11,12], foods containing PAHs are likely to contain their derivatives. However, we did not observe such a correlation when comparing the results between targeted and untargeted analysis, except for anthracene found in the smoked bacon (sample B1) that was contaminated with ATQ. Ant and ATQ have the same basic structure.

These findings could be explained considering that the PAHs derivatives formation can be enhanced in specific conditions (unknown in the analysed samples) and the possible endogenous different contamination of the samples prior to cooking/smoking due to the different bioaccumulative factor of OPAHs [67]. However, it should be taken into account that the non-target approach does not give a quantitative output, therefore these considerations could be affected by the sensibility and reproducibility of the detection of the tentatively identified PAHs.

## 4. Conclusions

The optimized chromatographic method provided a sensitive and specific quantification of NPAHs, OPAHs, and PAH4 by a single chromatographic run. Out of 15 samples, two samples exceeded the EU maximum level of ATQ (10 μg/kg) and the smoked bacon also exceeded the EU maximum limit of 2 μg/kg and 12 μg/kg for BaP and the sum of PAH4, respectively. The results of the analysed smoked samples indicated the scarce occurrence of NPAHs, while they highlighted the presence of OPAHs in much higher concentrations than PAHs. The non-targeted screening extended the investigation of other PAHs and their derivatives not commonly reported. The non-target screening tentatively identified additional OPAHs and other PAH derivatives to which the target quantitative analysis should be extend to in the future. The PAHs derivatives (OPAHs and NPAHs) can be directly formed during incomplete combustion processes but also by chemical, photochemical or biological reactions of PAHs and, in some cases, they can act as more powerful toxic agents, particularly for their enhanced carcinogenic effect compared to PAHs.

Nevertheless, limited literature is present as regard to the presence of these substances in processed products. The presented work, not only highlighted the presence in smoked food of many PAH derivatives not regulated yet, but also demonstrated the possibility to effectively monitor these contaminants of emerging concern. We presented one simultaneous analytical method for the regulated PAH4 and PAH derivatives, that allows for a faster production of more occurrence data needed for the further exposure evaluation and formation studies.

## Figures and Tables

**Table 1:** List of reported polycyclic aromatic hydrocarbons (PAHs), nitro-PAHs (NPAH) and oxy-PAHs (OPAH) with molecular formula, abbreviations, compound class, carcinogenic classification according to International Agency for Research on Cancer (IARC) and CAS number.

Compound	Molecular Formula	Abbreviation	Compound Class	IARC Group ^a^	CAS Num
1-Indanone	C_9_H_8_O	1-Ind-one	OPAH	*	83-33-0
1-Nitro-naphthalene	C_10_H_7_NO_2_	1NNap	NPAH	3	86-57-7
2-Nitro-naphthalene	C_10_H_7_NO_2_	2NNap	NPAH	3	581-89-5
Acenaphthylene	C_12_H_8_	Acy	PAH	*	208-96-8
4-Acenaphthylenol	C_12_H_8_O	4-Acy-ol	OPAH	*	111013-09-3
Acenaphthene	C_12_H_10_	Acn	PAH	3	83-32-9
5-Nitro-acenaphthene	C_12_H_9_NO_2_	5NAce	NPAH	2B	602-87-9
Fluorene	C_13_H_10_	Flu	PAH	3	86-73-7.
2-Nitro-fluorene	C_13_H_9_NO_2_	2NFlu	NPAH	2B	607-57-8
9-Fluorenone	C_13_H_8_O	9FLO	OPAH	*	486-25-9
2-Hydroxyfluorene	C_13_H_10_O	2-OH-Flu	OPAH	*	2443-58-5
Xantene	C_13_H_10_O	Xan	OPAH	*	92-83-1
4-Methylfluorene	C_14_H_12_	2-Me-Flu	Methylated-PAH	*	1556-99-6
Antracene	C_14_H_10_	Ant	PAH	3	1719-06-8.
9- Nitro-anthracene	C_14_H_9_NO_2_	9NAnt	NPAH	3	602-60-8
9,10-Antraquinone	C_14_H_8_O_2_	ATQ	OPAH	2B	84-65-1
Phenanthrene	C_14_H_10_	Phe	PAH	3	85-01-8
3- Nitro-phenanthrene	C_14_H_9_NO_2_	3NPhe	NPAH	*	17024-19-0
9-Nitro-phenanthrene	C_14_H_9_NO_2_	9NPhe	NPAH	*	954-46-1
4-Methylphenanthrene	C_15_H_10_	4-Me-Phe	Methylated-PAH	*	832-64-4
Fluoranthene	C_16_H_10_	Fla	PAH	3	206-44-0
3-Nitro-fluoranthene	C_16_H_9_NO_2_	3NFla	NPAH	3	892-21-7
Pyrene	C_16_H_10_	Pyr	PAH	3	129-00-0
1-Nitro-pyrene	C_16_H_9_NO_2_	1NPyr	NPAH	2A	5522-43-0
4-Nitro-pyrene	C_16_H_9_NO_2_	4NPyr	NPAH	2B	57835-92-4
1,3-Dinitro-pyrene	C_16_H_8_N_2_O_4_	1,3DNPyr	NPAH	2B	75321-20-9
1,6-Dinitro-pyrene	C_16_H_8_N_2_O_4_	1,6DNPyr	NPAH	2B	42397-64-8
1,8-Dinitro-pyrene	C_16_H_8_N_2_O_4_	1,8DNPyr	NPAH	2B	42397-65-9
Benz[a]anthracene	C_18_H_12_	BaA	PAH	2B	56-55-3
7-Nitro-benz[*a*]anthracene	C_18_H_11_NO_2_	7NBaA	NPAH	3	20268-51-3
Chrysene	C_18_H_12_	Chr	PAH	2B	218-01-9
6- Nitro-chrysene	C_18_H_11_NO_2_	6NChr	NPAH	2A	7496-02-8
Benzo[*b*]fluoranthene	C_20_H_12_	BbF	PAH	2B	205-99-2
Benzo[a]pyrene	C_20_H_12_	BaP	PAH	1	50-32-8
6-Nitro-benzo[*a*]pyrene	C_20_H_11_NO_2_	6NBaP	NPAH	3	63041-90-7

^a^ [13,14,15,16,17]. Classification according to IARC in group 1: carcinogenic to humans, 2A: probable carcinogenic, 2B: possible carcinogenic, 3: not classifiable as carcinogenic whereas compounds marked with * indicate that no IARC classification is available. N/A indicates not applicable.

**Table 2:** Analysed compounds with retention times and *m*/*z*-values for quantification with the in-laboratory reproducibility RSD_r_ (N = 8) repeatability RSD_R,_ matrix effects (N = 3) and the total recovery (%) mean values obtained from the three concentration levels validated. The method limit of detection (LOD) and limit of quantification (LOQ) were expressed as µg/kg. “-” are not included or not relevant. Full names can be seen in Table 1.

Compounds	QTOF (*m*/*z*)	Retention Time (Min)	RSD_R_	RSD_r_	Total Recovery (%)	LOD (µg/kg)	LOQ (µg/kg)	Matrix Effect (%)
1NNap-d_7_	134.097	8.61	-	-	-	-	-	-
1NNap	127.053	8.65	9	6	101	0.70	1.40	93
2NNap	127.053	9.08	8	8	93	0.38	0.75	92
9FLO	180.057	10.15	14	11	96	0.80	1.60	113
ATQ	208.052	14.13	13	9	64	0.45	0.89	90
5NAce	152.061	14.80	8	8	94	0.43	0.86	-
2NFlu-d_9_	174.126	16.42	-	-	-	-	-	-
2NFlu	165.069	16.56	8	6	101	0.54	1.08	113
9NAnt-d_9_	232.118	16.78	-	-	-	-	-	-
9NAnt	176.062	16.87	6	5	96	0.29	0.58	100
9NPhe	176.062	18.08	6	5	97	0.19	0.39	114
3NPhe	223.062	18.73	8	6	98	0.49	0.98	114
3NFla-d_9_	226.143	22.00	-	-	-	-	-	-
3NFla	247.062	22.06	6	5	93	0.69	1.38	90
4NPyr	247.062	22.26	10	6	103	0.55	1.11	97
1NPyr-d_9_	226.142	22.73	-	-	-	-	-	-
1NPyr	247.062	22.79	9	4	99	0.59	1.18	103
6NChr-d_11_	284.145	24.11	-	-	-	-	-	-
7NBaA	215.086	24.59	10	9	107	0.44	0.88	98
6NChr	273.079	25.61	6	4	97	0.47	0.94	98
1,3DNPyr *	200.061	26.83	18	8	70	1.55	3.10	82
1,6DNpyr *	292.046	28.19	32	8	91	3.46	6.93	106
1,8DNpyr *	292.046	28.56	70	25	-	3.21	6.42	90
6NBaP	297.079	30.40	13	13	88	0.35	0.71	90

* only including spike levels passing the validation.

**Table 3:** Concentration of the polycyclic aromatic hydrocarbon (PAH), nitro-PAH (NPAHs), oxy-PAH (OPAHs) expressed as µg/kg in 11 commercially smoked fish, one commercially smoked bacon (pork) sample, and four home cooked cod samples. The presented values are associated to the averaged method RSD_R_ associated to each compound reported in Table S3 (total RSD_R_). The values in bold are sum of nitrated PAHs (ΣNPAH), oxygenated PAHs (ΣOPAH) and PAH (ΣPAH4).

Fish/Meat	Cod (C1)	Cod (C2)	Cod (C3)	Cod (C4)	Halibut (HA1)	Halibut (HA2)	Herring (HE1)	Herring (HE2)	Mackerel (MA1)	Mackerel (MA2)	Mackerel (MA3)	Salmon (SA1)	Salmon (SA2)	Salmon (SA3)	Salmon (SA4)	Bacon (B1)
Smoking Process	Boiled	Pan Fried	Hot (200 °C)	Hot (360 °C)	Traditional	Hot	Hot	Hot	Hot	Hot	Hot	Hot	Cold	Cold	Hot	Hot
1NNap	nd	nd	nd	nd	nd	nd	5.64	nd	nd	nd	nd	nd	nd	0.46 *	nd	nd
2NNap	nd	nd	nd	nd	nd	nd	nd	nd	nd	nd	0.70 *	nd	nd	nd	nd	nd
5NAce	nd	nd	nd	nd	nd	nd	nd	nd	nd	nd	0.43 *	nd	nd	nd	nd	0.86 *
2NFlu	nd	nd	nd	nd	nd	nd	nd	nd	nd	nd	nd	nd	nd	nd	nd	nd
9NAnt	nd	nd	nd	nd	nd	nd	nd	nd	nd	nd	nd	nd	nd	nd	nd	1.02
9NPhe	nd	nd	nd	nd	nd	nd	nd	nd	nd	nd	nd	nd	nd	nd	nd	nd
3NPhe	nd	nd	nd	nd	nd	nd	nd	nd	nd	nd	nd	nd	nd	nd	nd	nd
3NFla	nd	nd	nd	nd	nd	nd	nd	nd	nd	nd	nd	nd	nd	nd	nd	nd
4NPyr	nd	nd	nd	nd	nd	nd	nd	nd	nd	nd	nd	0.16 *	nd	nd	nd	nd
1NPyr	nd	nd	nd	nd	nd	nd	nd	nd	nd	nd	nd	nd	nd	nd	nd	nd
7NBaA	nd	nd	nd	nd	nd	nd	nd	nd	nd	nd	nd	nd	nd	nd	nd	nd
6NChr	nd	nd	nd	nd	nd	nd	nd	nd	nd	nd	nd	nd	nd	nd	nd	nd
1,3DNPyr	nd	nd	nd	nd	nd	nd	nd	nd	nd	nd	nd	nd	nd	nd	nd	nd
1,6DNPyr	nd	nd	nd	nd	nd	nd	nd	nd	nd	nd	nd	nd	nd	nd	nd	nd
6NBaP	nd	nd	nd	nd	nd	nd	nd	nd	nd	nd	nd	nd	nd	nd	nd	nd
**ΣNPAH**	**nd**	**nd**	**nd**	**nd**	**nd**	**nd**	**5.64**	**nd**	**nd**	**nd**	**1.13**	**0.16**	**nd**	**0.46**	**nd**	**1.88**
9FLO	3.40	4.92	14.83	23.41	13.36	10.17	15.56	21.25	nd	nd	8.20	7.24	1.14 *	2.02	15.82	162.12 **
ATQ	2.31	2.90	6.63	10.54	1.50	2.54	8.63	4.32	nd	nd	1.35	1.50	0.97	0.82 *	5.92	25.63
**ΣOPAH**	**5.71**	**7.82**	**21.47**	**33.95**	**14.86**	**12.71**	**24.19**	**25.58**	**nd**	**nd**	**9.56**	**8.73**	**2.11**	**2.84**	**21.75**	**187.74**
BaA	nd	nd	0.10 *	0.04 *	nd	nd	nd	0.38	nd	nd	nd	nd	nd	nd	nd	13.86
CHR	nd	nd	0.24	0.13 *	nd	nd	nd	0.21 *	0.67	nd	nd	nd	nd	nd	nd	14.39
BbF	nd	nd	nd	nd	nd	nd	nd	nd	nd	nd	nd	nd	nd	nd	nd	4.78
BaP	nd	nd	nd	nd	nd	nd	nd	nd	nd	nd	nd	nd	nd	nd	nd	5.30
**ΣPAH4**	**nd**	**nd**	**0.34**	**0.17**	**nd**	**nd**	**nd**	**0.59**	**0.67**	**nd**	**nd**	**nd**	**nd**	**nd**	**nd**	**38.33**

* indicate values below LOQ but above LOD and is included in the sum, nd indicates not detected and is summed as zero, ** indicate values out of calibration range.

**Table 4:** List of tentatively identified substances (PAH) at a confidence level of 2a (match with a library spectrum) based on Schymanski et al., 2014 [63] in the processed fish products.

S No.	Compound	Mol. Formula	*m*/*z*	Rt (Min)	Sample Name
1	1-Ind-one	C_9_H_8_O	132.057	6.39	SA1, SA2, SA3, SA4, HE1, HE2, HA1, MA2, B1
2	Acy	C_12_H_8_	152.063	7.23	HE2, MA2
3	Acn	C_12_H_10_	153.070	7.40	HE2
4	4-Acy-ol	C_12_H_8_O	168.059	7.58	B1
5	Flu	C_13_H_10_	166.077	8.16	HA1, HE2, B1, SA1, MA1
6	2-OH-Flu	C_13_H_10_O	181.066	8.46	B1
7	Xan	C_13_H_10_O	182.074	8.47	HE2, MA1
8	2-Me-Flu	C_14_H_12_	165.071	9.19	HE2
9	Phe	C_14_H_10_	178.079	10.60	C2, HE2, MA1, HA1
10	Ant	C_14_H_10_	178.079	10.73	B1
11	4-Me-Phe	C_15_H_12_	192.095	12.07	B1
12	Fla	C_16_H_10_	202.08	15.28	HE2, B1, MA1
13	Pyr	C_16_H_10_	202.079	16.45	HE2, B1

SA: Salmon, HE: Herring, B: Bacon, MA: Mackerel, C: Cod, HA: Halibut.

## Data Availability

The date are available from the corresponding author.

## Supplementary Materials

The following supporting information can be downloaded at: https://www.mdpi.com/article/10.3390/foods11162446/s1, Table S1. Schematic description of the samples collected. The fat determination was gravimetrically determined of ASE extracts as described by Lund et al., 2009; Table S2. Recoveries (%) and coefficient of variation (CV%) in brackets for evaporation at different evaporation conditions with either rotavapor or nitrogen flow(N2-flow) at 35 °C or 40 °C as well as overall extraction recoveries and CV% for original and final optimised protocol (N = 3); Table S3. Results for single spike levels (N = 8) for NPAH and OPAH intra-laboratory reproducibility, intra-laboratory repeatability (mean, RSD and HorRat values) and the total recovery.
