# Supplementary material for: Detection of Nitrated, Oxygenated and Hydrogenated Polycyclic Aromatic Compounds in Smoked Fish and Meat Products
Source: Foods. 2022 Aug 13;11(16):2446. doi: 10.3390/foods11162446 (PMC9407348; doi:10.3390/foods11162446)
Supplement: Supplementary file 1 [file foods-11-02446-s001.zip › foods-1830211-supplementary.pdf]

Table S1. Schematic description of the samples collected. The fat determination was gravimetrically determined of ASE extracts as described by Lund et al. 2009.

Sample code	Sample type	Smoking process	Retailer	Fat %
C0	Cod	raw (validation)	supermarket	0.5
C2	Cod	pan fried	supermarket	1.4
C3	Cod	hot smoked (exp 1)	supermarket	1.5
C4	Cod	hot smoked (exp 2)	supermarket	1.4
HA1	Halibut	traditional smoked	supermarket	23.5
HA2	Halibut	hot smoked	fish monger	14.2
HE1	Herring	hot smoked	fish monger	24.0
HE2	Herring	hot smoked	fish monger	21.5
MA1	Mackerel	hot smoked	fish monger	24.1
MA2	Mackerel	hot smoked	supermarket	24.3
MA3	Mackerel	hot smoked	fish monger	24.1
SA1	Salmon	hot smoked	fish monger	19.4
SA2	Salmon	cold smoked	supermarket	4.1
SA3	Salmon	cold smoked (beech chips)	supermarket	18.7
SA4	Salmon	hot smoked	fish monger	21.0
B1	Bacon	hot smoked	Polish market	22.5

Table S2. Recoveries (%) and coefficient of variation (CV%) in brackets for evaporation at different evaporation conditions with either rotavapor or nitrogen flow(N2-flow) at 35°C or 40 °C as well as overall extraction recoveries and CV% for original and final optimised protocol (N=3).

	**Rotavapor**		**N_2-flow**		**Original protocol**		**Final protocol**	
**Compound**	**40°C**	**35 °C**	**40°C**	**35 °C**	**Recovery %**	**CV%**	**Recovery %**	**CV%**
1NNap	61 (2)	47 (3)	49 (21)	64 (3)	35	13	61	7
2NNap	63 (5)	47 (6)	51 (14)	66 (5)	30	20	61	6
9-FLO	65 (5)	52 (5)	54 (19)	66 (9)	36	15	65	16
ATQ	60 (2)	69 (7)	45 (14)	60 (5)	37	16	68	13
2NFlu	67 (3)	75 (6)	52 (16)	75 (3)	38	12	71	15
9NAnt	60 (7)	71 (6)	49 (19)	74 (8)	42	20	67	9
9NPhe	71 (1)	69 (3)	57 (20)	85 (9)	46	12	68	14
3NPhe	65 (5)	67 (9)	52 (15)	88 (7)	42	14	68	13
3NFla	74 (4)	72 (7)	47 (27)	74 (11)	34	16	74	14
4NPyr	60 (15)	82 (6)	48 (15)	75 (8)	34	17	82	11
1NPyr	67 (7)	75 (3)	48 (29)	90 (3)	40	17	72	12
7NBaA	55 (18)	77 (4)	48 (9)	88 (10)	40	17	76	11
6NChr	64 (13)	75 (4)	51 (14)	91 (6)	41	15	70	13
1,3DNPyr	63 (16)	54 (9)	-	88 (5)	18	25	28	27
1,6DNPyr	56 (19)	44 (18)	-	86 (5)	16	25	32	23
1,8DNPyr	63 (15)	48 (30)	-	96 (16)	8	23	20	34
6NBaP	53 (28)	63 (7)	24 (15)	82 (6)	30	20	55	8
Average	63	64	48	79	33	**-**	68	**-**

- *not determined*

Table S3. Results for single spike levels (N= 8) for NPAH and OPAH intra-laboratory reproducibility, intra-laboratory repeatability (mean, RSD and HorRat values) and the total recovery each spike level.

		In-laboratory reproducibility			Repeatability			
	Spike level (µg/g)	mean	RDS_R (%)	$HorRat_R$	mean	RDS_r (%)	$HorRat_r$	Total Recovery (%)
1NNap	10	10	7	0.32	10	6	0.24	98
	5	5	7	0.34	5	5	0.23	101
	2	2	11	0.51	2	7	0.29	103
	Total		*9*			*6*		*101*
2NNap	10	10	8	0.39	10	8	0.37	95
	5	5	9	0.40	5	9	0.38	95
	2	2	7	0.32	2	7	0.30	88
	Total		*8*			*8*		*93*
9FLO	10	11	12	0.56	11	6	0.26	105
	5	5	14	0.65	5	12	0.51	94
	2	2	15	0.67	2	14	0.59	90
	Total		*14*			*11*		*96*
ATQ	11	7	11	0.52	7	11	0.47	63
	5	3	16	0.73	3	7	0.31	63
	2	1	10	0.48	1	7	0.30	66
	Total		*13*			*9*		*64*
5NAce	10	10	6	0.27	10	6	0.25	96
	5	5	10	0.47	5	10	0.45	97
	2	2	8	0.36	2	8	0.35	90
	Total		*8*			*8*		*94*
2NFlu	10	10	6	0.25	10	4	0.18	99
	5	5	10	0.45	5	8	0.36	103
	2	2	9	0.40	2	3	0.13	102
	Total		*8*			*6*		*101*
9NAnt	10	10	6	0.27	10	4	0.18	96
	5	5	6	0.27	5	6	0.25	97
	2	2	5	0.23	2	4	0.18	94
	Total		*6*			*5*		*96*
9NPhe	10	10	4	0.19	10	2	0.10	97
	5	5	8	0.36	5	8	0.35	99
	2	2	3	0.15	2	3	0.15	96
	Total		*6*			*5*		*97*
3NPhe	10	10	6	0.29	10	3	0.14	102
	5	5	9	0.42	5	8	0.34	93
	2	2	8	0.37	2	5	0.23	100
	Total		*8*			*6*		*98*
3NFla	20	20	5	0.23	20	5	0.20	100
	10	9	7	0.31	9	3	0.15	95
	4	3	7	0.31	3	7	0.30	84
	Total		*6*			*5*		*93*
4NPyr	9	10	7	0.30	10	7	0.29	116
	5	4	12	0.54	4	5	0.23	91
	2	2	10	0.45	2	7	0.29	103
	Total		*10*			*6*		*103*
1NPyr	10	11	4	0.18	11	4	0.16	106
	5	5	12	0.52	5	4	0.16	100
	2	2	11	0.48	2	5	0.22	92

	Total		*9*			*4*		*99*
7NBaA	10	10	4	0.17	10	3	0.13	104
	5	5	15	0.67	5	15	0.64	106
	2	2	7	0.30	2	7	0.28	110
	Total		*10*			*9*		*107*
6NChr	10	10	3	0.13	10	3	0.13	97
	5	5	6	0.28	5	4	0.19	96
	2	2	8	0.37	2	6	0.25	96
	Total		*6*			*4*		*97*
1,3DNPyr	20	9	48	2.20	9	19	0.82	43
	10	3	87	3.94	3	14	0.59	31
	4	3	18	0.84	3	8	0.33	70
	Total		*58*			*14*		*48*
1,6DNpyr	20	12	47	2.12	12	17	0.76	60
	10	4	69	3.13	4	36	1.56	37
	4	4	32	1.44	4	8	0.33	91
	Total		*51*			*23*		*62*
1,8DNpyr	20	7	82	3.74	7	31	1.33	34
	10	3	74	3.35	3	30	1.33	27
	4	2	49	2.21	2	6	0.28	55
	Total		*70*			*25*		*39*
6NBaP	10	9	13	0.60	9	13	0.56	92
	5	4	18	0.80	4	17	0.76	82
	2	2	6	0.29	2	6	0.28	92
	Total		*13*			*13*		*88*
